# A retrospective study of venous thromboembolism in acute leukemia patients treated at the University of Texas MD Anderson Cancer Center

**DOI:** 10.1002/cam4.332

**Published:** 2014-12-08

**Authors:** Khanh Vu, Nhiem V Luong, Julie Hubbard, Ali Zalpour, Stefan Faderl, Deborah A Thomas, Daisy Yang, Hagop Kantarjian, Michael H Kroll

**Affiliations:** 1 Department of General Internal Medicine, The University of Texas MD Anderson Cancer CenterHouston, Texas; 2 The University of Texas Health Science Center at HoustonHouston, Texas; 3 Annapolis Oncology CenterAnnapolis, Maryland; 4 Department of Pharmacy, The University of Texas MD Anderson Cancer CenterHouston, Texas; 5 Department of Leukemia, The University of Texas MD Anderson Cancer CenterHouston, Texas; 6 Department of Pulmonary Medicine, Section of Thrombosis and Benign Hematology, The University of Texas MD Anderson Cancer CenterHouston, Texas

**Keywords:** Acute leukemia, anticoagulation, cancer, thrombosis, venous thromboembolism

## Abstract

The purpose was to determine the incidence and prevalence of venous thromboembolism (VTE) in acute leukemia patients from our institution. We conducted a retrospective study on newly diagnosed acute leukemia patients who presented at our institution from November 1999 to May 2005. Descriptive statistics and cross-tabulation were used to describe patient characteristics. Measures of morbidity were used to address VTE risk. Chi-square testing, Fisher's exact testing, Mann–Whitney analyses, or median testing were used to determine between-group differences. Data analyses were conducted using Stata version 11 (Stata Corp., College Station, TX). Two hundred and ninety-nine patients with acute lymphoblastic leukemia (ALL) and 996 patients with acute myeloid leukemia (AML) were included. After excluding patients diagnosed with VTE prior to or at the time of leukemia diagnosis, during the mean time follow-up period of 2.5 years (range: 0.0025–10.3 years), the overall incidence rate of VTE was 3.7 per 100 person-years: 4.2 per 100 person-years for ALL and 3.4 per 100 person-years for AML. Among all patients, the majority (80.6%) developed VTE within 12 months after diagnosis and during thrombocytopenia. The most common VTE was central venous catheter (CVC)-associated upper-extremity deep venous thrombosis. Pulmonary embolism occurred in 15% of ALL patients and 8% of AML patients. VTE recurred in 20.7% of ALL patients and 18.6% of AML patients. VTE occurs frequently in patients with acute leukemia. Studies are needed to identify risk factors for the development and recurrence of VTE among patients with acute leukemia and to establish more effective methods for preventing and treating VTEs in leukemia patients who have thrombocytopenia and/or CVC.

## Introduction

The association between cancer and thrombosis is established 1. Venous thromboembolism (VTE) can precede a diagnosis of cancer, be the presenting symptom of cancer, or develop during cancer treatment. The risk of VTE is four to seven times higher in cancer patients than in patients without cancer 2, and about 20% of VTEs are diagnosed in cancer patients. Symptomatic and asymptomatic VTE occur in cancer patients at rates of 15% and 50%, respectively, and up to 50% of cancer patients have VTE at autopsy 3. Pulmonary embolism (PE) is the attributed cause of death in up to 3.5% of cancer patients 4,5 and may be an unattributed cause of death in a similar number of cancer patients 6. VTE prevention is an essential component of the care of hospitalized cancer patients 7.

Cancer confers hypercoagulability through multiple mechanisms, including the release of tumor procoagulants; reduced levels of natural anticoagulants; decreased fibrinolytic activity; increased coagulation factors; increased platelet activation; and mucin- and cytokine-induced endothelial cell tissue factor expression 8,9. Acute leukemias are prothrombotic because of the release of leukemic cell procoagulant activity 10, particularly after treatment-induced leukemia cell killing 11.

The California Cancer Registry (CCR) reported that 3.7% of patients with acute myeloid leukemia (AML), 2.7% of patients with chronic lymphocytic leukemia, 2.6% of patients with acute lymphoblastic leukemia (ALL), and 1.5% of patients with chronic myelogenous leukemia developed VTE within 1 year after diagnosis 12. Among patients with acute leukemia, the annual incidence of VTE is reportedly highest in patients with acute promyelocytic leukemia (APL: 6.1–42.8%) and lowest in patients with ALL (2.1–13.0%) 13.

Clinical risk factors for VTE include previous VTE, chemotherapy, central venous catheter (CVCs), comorbid conditions, a body mass index >30, immobility, and/or the use of erythropoiesis-stimulating agents 14. For example, in one study of 343 ALL patients, VTE occurred in 11.1% of patients who received l-asparaginase (a drug causing hypercoagulability because it inhibits fibrinolysis) but was observed in only 2.0% of those who did not receive it 15. A multivariate analysis of risk factors for deep vein thrombosis (DVT) or PE within 1 year of diagnosis in AML patients revealed that the hazard ration (HR) for VTE was higher for patients with CVCs versus those without (HR = 1.5) 15.

Cancer-associated VTE is a clinical marker of more aggressive and less treatable cancers, and is associated with increased cancer-related mortality. According to CCR data, 80% of cancer patients with VTE died within 6 months of VTE diagnosis 16. A prospective analysis revealed that 40% of cancer patients with VTE died within 6 months of VTE onset, with 90% of these deaths attributed to cancer progression (4.5% were attributed to PE) 17. A recent review of the CCR cross-referenced with the California Discharge Database revealed that VTE was associated with a higher risk of death in ALL but not AML patients (HR = 1.4) 18, probably because high early mortality in AML is an overwhelming competing risk 19.

To determine the incidence, prevalence, and clinical impact of VTE in acute leukemia patients, we analyzed clinical data from 1295 patients admitted to our institution during a 5-year period.

## Methods

### Patient population

The study was approved by the MD Anderson Institutional Review Board. We reviewed the charts of consecutive patients with newly diagnosed ALL (including Burkitt's lymphoma and lymphoblastic leukemia) or AML who presented to The University of Texas MD Anderson Cancer Center (UTMDACC) from November 1999 to May 2005. Analyses were from admission date through 31 May 2010, at that time 26.2% were alive, 73.7% were dead, and <0.1% lost to follow-up.

Patients' demographics, leukemia diagnosis, comorbidities, chemotherapy, and baseline laboratory data were collected. Screening for hypercoagulability was not done. For those with new VTE, we tabulated location of VTE, radiographic modality of diagnosis, synchronous laboratory data, VTE treatment, and date of last follow-up or death. Patients' medication information was collected from the chart review and a database maintained by UTMDACC's Medical Informatics. Data were collected from the date of admission (between November 1999 and May 2005) until 31 May 2010, last follow-up or death, whichever came first.

### Study design

The rate of VTE was the principal study outcome. VTE was defined as a thrombosis in the pulmonary vasculature (PE) or a DVT in the upper extremities (axillary, subclavian, and/or jugular veins) or lower extremities (femoral, popliteal, superficial femoral, and/or iliac veins). Catheter-associated thrombosis was defined as a DVT in the upper extremities or lower extremities identified within 3 months of catheter insertion. Diagnostic imaging used to confirm VTE included venography, Doppler ultrasonography, pulmonary ventilation-perfusion scan, computed tomography (CT), and/or CT angiography. Patients with synchronous PE and DVT were classified as having a PE with specification of the location of the DVT. VTE recurrence was defined by an acute VTE in a different vascular bed.

### Statistical analysis

Analyses of the ALL and AML groups were performed separately. Descriptive statistics such as frequencies, means, medians, and cross-tabulation were used for patient characteristics. Between-group differences were evaluated using the chi-square test or Fisher's exact test for categorical data or a Mann–Whitney test or median test for continuous data. Risk factors associated with the development of VTE were analyzed by logistic regression models. Hosmer and Lemeshow stepwise strategies were applied for model building: potential independent variables with *P*-value <0.25 were included in the initial full model. Prevalence, initial episode incidence, and recurrence rate of VTE were determined. Calculation of prevalence was based on the number of patients who had VTE prior to their admission to our institution plus the number of patients who had VTE incidentally discovered at the time of leukemia diagnosis plus those who developed VTE during the observation period. To determine the rate of developing VTE during the treatment period, the incidence density of VTE was estimated; patients who had VTE diagnosed prior to or at the time of their diagnosis were excluded. Data analyses were performed using the Stata software program (version 12; STATA Corp., College Station, TX). *P* values less than 0.05 (two-tailed) were considered statistically significant.

## Results

We identified 299 patients with ALL and 996 patients with AML, 73 of whom had APL. Baseline characteristics of these 1295 patients are presented in Table1. VTE was diagnosed in 53 ALL patients (17.7%) and 86 AML patients (8.6%), for an overall prevalence of 10.7%. Compared with ALL patients who did not develop VTE, ALL patients who developed VTE had a significantly higher median age (48 years versus 42 years, *P* = 0.008) and a significantly lower median serum creatinine level (0.8 mg/dL versus 0.9 mg/dL, *P* = 0.005). No such differences were detected in AML patients.

**Table 1:** Patient characteristics

	ALL (*N *=* *299)			AML (*N* = 996)		
Characteristic	VTE (*N* = 53)	No VTE (*N* = 246)	*P*	VTE (*N* = 86)	No VTE (*N* = 910)	*P*
Age, years	48 (19–75)	42 (15–83)	0.008^1^	57.5 (18–81)	60 (14–89)	0.28
BMI, kg/m^2^	26.2 (15.2–44.9)	25.9 (15.6–51.6)	0.88	27.2 (17.3–58.1)	26.7 (14.3–73.0)	0.78
Leukocyte count, x10^9^/L	7.3 (0.9–281.5)	6.8 (0.3–602.5)	0.77	4.8 (0.4–237.5)	7.0 (0.4–390)	0.38
Hemoglobin count, mg/dL	9.6 (4.3–13.8)	9.2 (3.5–16.7)	0.43	8.4 (4.0–12.6)	7.9 (2.5–14.4)	0.23
Platelet count, x10^9^/L	87 (16–404)	60 (3–668)	0.13	63.0 (8–762)	47.0 (3–676)	0.06
Serum LDH level, IU/L	807.5 (188–33175)	1061 (172–42001)	0.23	855.0 (303–9267)	872.5 (190–25980)	0.67
Serum fibrinogen level, mg/dL	448 (77–995)	394 (49–879)	0.12	392.0 (77–701)	387.0 (81–1000)	0.67
PT, seconds	12.4 (10.9–18.4)	12.7 (10.6–23.4)	0.16	12.9 (11–23.5)	13.2 (10–24.5)	0.15
PTT, seconds	27.3 (20.5–37.4)	27.6 (18.5–56.4)	0.31	27.4 (21.3–984)	27.8 (19.3–297)	0.48
Serum albumin level, mg/dL	3.3 (1.8–4.6)	3.4 (1.8–5.0)	0.26	3.4 (1.9–4.6)	3.4 (1.4–5.3)	0.36
Serum creatinine level, mg/dL	0.8 (0.5–1.8)	0.9 (0.4–6.2)	0.005^1^	0.9 (0.5–4.5)	0.9 (0.3–6.8)	0.64
Serum glucose level, mg/dL	97 (69–263)	106 (9–469)	0.12	104.0 (76–222)	105.0 (42–584)	0.74

All data are the median value (range) unless otherwise indicated. ALL, acute lymphoblastic leukemia; AML, acute myeloid leukemia; BMI, body mass index; LDH, lactate dehydrogenase; PT, prothrombin time; PTT, partial thromboplastin time VTE, venous thromboembolism.

1 Statistically significant.

No patient who developed VTE received any anticoagulation therapy, including pharmacological VTE prophylaxis, in the month before VTE onset. Among those who did not develop VTE, 3/246 with ALL and 3/910 with AML received pharmacological VTE prophylaxis at some point during their leukemia treatment program; and 5/246 (2%) with ALL and 6/910 (0.7%) with AML were receiving anticoagulation for other indications (atrial fibrillation, chronic VTE, cerebrovascular ischemia, or heart failure).

The rates of VTE according to patient characteristics are given in Table2. Among ALL patients, 14/49 (28.6%) with Philadelphia chromosome-positive disease developed VTE. Among the AML patients, 8/73 (11%) with APL developed VTE. The majority of all patients were Caucasian, including those who developed VTE: 67.9% of ALL patients and 84.9% of AML patients. African-American patients with ALL developed more VTE compared with other ethnicities; the percentages were 33.3%, 20.3%, and 12.5% among African-American, Caucasian, and Hispanic patients, respectively (*P* = 0.04). Older ALL patients developed more VTE: the percentage of patients with VTE was 11.8% among those who aged ≤39 years versus 25.2% and 17.4% among those 40–59 and ≥60 years, respectively (*P* = 0.03). Younger AML patients (≤39 years old) had a higher number of VTEs (14.8%) compared with 8.1% and 7.4% in the older age groups of 40–59 and ≥60 years, respectively (*P* = 0.04). The percentage of patients with a prior history of VTE was significantly higher among those who developed VTE. Among ALL patients, 11.3% of the patients who developed VTE had a history of VTE, whereas only 1.6% of the patients without VTE had a history of VTE (*P* = 0.003); among AML patients, 7.0% of the patients who developed VTE had a history of VTE, whereas only 2.1% of patients without VTE had such a history (*P* = 0.02). For patients with AML who received an erythropoiesis-stimulating agent (ESA), the percentage developing VTE (46.5%) was significantly higher than those not developing VTE (30.2%, *P* = 0.003).

**Table 2:** Prevalence of venous thromboembolism (VTE) by patient characteristics

	ALL (*N* = 299)			AML (N = 996)		
Characteristic	VTE (N = 53 [17.7%])	No VTE (N = 246 [82.3%])	*P*	VTE (N = 86 [8.6%])	No VTE (N = 910 [91.4%])	*P*
Diagnosis/Subtypes						
Philadelphia-negative ALL	26 (49.1)	144 (58.6)	0.27			
Philadelphia-positive ALL	14 (26.4)	35 (14.2)				
Burkitt's leukemia/lymphoma	9 (17.0)	47 (19.1)				
T-lymphoblastic lymphoma	4 (7.5)	14 (5.7)				
Others	0 (0.0)	6 (2.4)				
Non-APL AML				78 (90.7)	845 (92.9)	0.46
APL				8 (9.3)	65 (7.1)	
Age, years						
≤39	15 (28.3)	112 (45.5)	0.03^1^	19 (22.1)	109 (12.0)	0.04^1^
40–59	26 (49.1)	77 (31.3)		29 (33.7)	328 (36.0)	
≥60	12 (22.6)	57 (23.2)		38 (44.2)	473 (52.0)	
Ethnicity						
Caucasian	36 (67.9)	141 (57.3)	0.04^1^	73 (84.9)	733 (80.6)	0.45
African-American	6 (11.3)	12 (4.9)		4 (4.6)	64 (7.0)	
Hispanic	11 (20.8)	77 (31.3)		9 (10.5)	94 (10.3)	
Other	0 (0.0)	16 (6.5)		0 (0.0)	19 (2.1)	
Sex						
Male	28 (52.8)	155 (63.0)	0.21	51 (59.3)	513 (56.4)	0.65
Female	25 (47.2)	91 (37.0)		35 (40.7)	397 (43.6)	
Obesity (BMI ≥ 30), kg/m^2^	14 (26.4)	68 (27.6)	0.81	27 (31.4)	271 (29.8)	0.93
Nonsmoker	30 (56.6)	146 (59.4)	0.69	54 (62.8)	584 (64.2)	0.41
Erythropoietin therapy	10 (18.9)	63 (25.6)	0.37	40 (46.5)	275 (30.2)	0.003^1^
Hormone replacement therapy/contraceptives						
Yes	18 (33.9)	51 (20.7)	0.07	22 (25.6)	151 (16.6)	0.44
No (women)	10 (18.8)	42 (17.1)		14 (16.3)	350 (38.5)	
No (men with prostate cancer)	2 (3.9)	3 (1.2)		43 (50.0)	33 (3.6)	
Unknown	23 (43.4)	150 (61.0)		7 (8.1)	376 (41.3)	
History of malignancy	5 (9.4)	25 (10.2)	0.55	21 (24.4)	200 (22.0)	0.58
History of VTE	6 (11.3)	4 (1.6)	0.003^1^	6 (7.0)	19 (2.1)	0.02^1^

All data are no. of patients (%) unless otherwise indicated. ALL, acute lymphoblastic leukemia; AML, acute myeloid leukemia; APL, acute promyelocytic leukemia; BMI, body mass index.

1 Statistically significant.

Results from multivariable logistic models are both corroborative and revealing. Among patients diagnosed with ALL, identified risk factors were baseline platelet count of 50–99 × 10^9^/L (adjusted odds ratio [aOR]: 3.7, 95% CI: 1.45–9.43), Philadelphia chromosome positivity (aOR: 2.8, 95%), age >40 years (aOR: 2.7, 95% CI: 1.21–6.21), the presence of an additional hematologic malignancy (myeloma, lymphoma, or AML; aOR: 8.3, 95% CI: 1.07–63.88), and, for women, hormonal therapy (aOR: 2.4, 95% CI: 1.07–5.24). Among AML patients, risk factors were maleness (aOR: 1.9, 95% CI: 1.02–3.7), liver disease (aOR: 3.7, 95% CI: 1.14–12.1), use of an ESA (aOR: 2.4, 95% CI: 1.5–4.05), and, for women, hormonal therapy (aOR: 2.6, 95% CI: 1.3–5.4).

**Figure 1:**
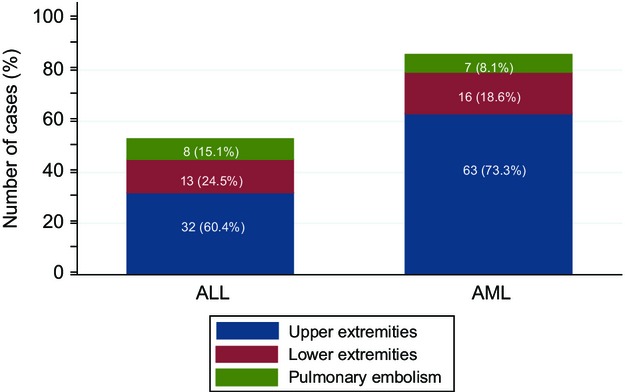
VTE distribution by anatomic location according to leukemia type. VTE, venous thromboembolism.

The location of thromboses in ALL patients is shown in Figure1. Of the 53 VTEs, 32 (60.4%) were in upper extremities. Of these 32, 28 (87.5%) were CVC related. Eight ALL patients (15.1%) developed PEs, of which four were associated with a lower extremity DVT and two were associated with an upper-extremity DVT; the source of two PEs could not be identified.

**Figure 2:**
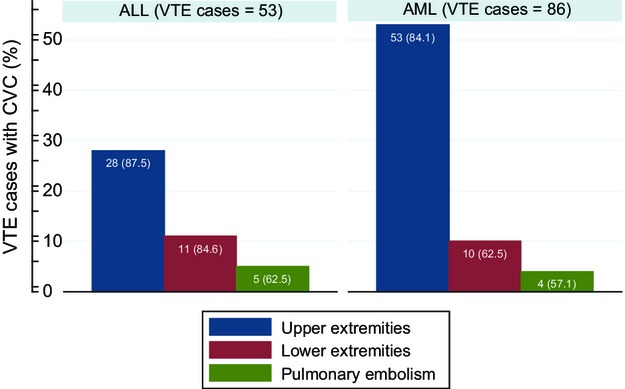
CVC-VTE event by anatomic location. CVC, central venous catheter; VTE, venous thromboembolism.

The location of thromboses in AML patients is shown in Figure2. Of the 86 VTEs, 63 (73.3%) were in upper extremities. Of the 63, 53 (84.1%) were CVC related. Seven AML patients (8.1%) had PEs, only one of which was associated with an identifiable DVT (of the lower extremity), and 16 (18.6%) had lower extremity DVT. There was no difference in the distribution of VTEs between ALL and AML patients (*P* = 0.245).

**Figure 3:**
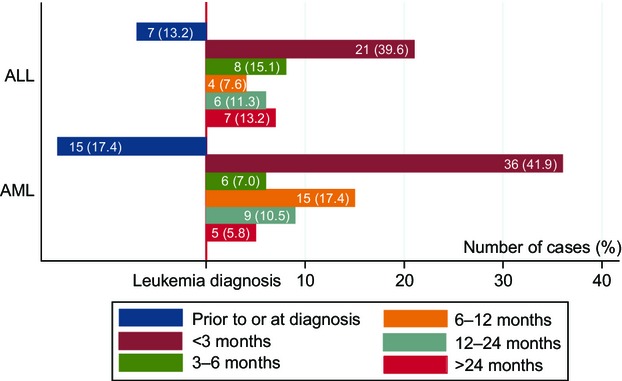
Time to VTE event by leukemia type since leukemia diagnosis. VTE, venous thromboembolism.

Most patients developed VTE within 3 months of diagnosis (Fig.3). Twenty-two patients (7 ALL patients and 15 AML patients) had VTE prior to or at the time of diagnosis. Among all VTEs, 1/53 and 3/86 were diagnosed within 1 month before the diagnosis of AML and ALL, respectively; and 6/53 and 12/86 with were diagnosed synchronously with AML and ALL, respectively. Excluding these patients, during the mean time follow-up period of 2.5 years (range: 0.0025–10.3 years), the overall incidence rate of VTE was 3.7 per 100 person-years: 4.2 per 100 person-years for ALL and 3.4 per 100 person-years for AML.

**Figure 4:**
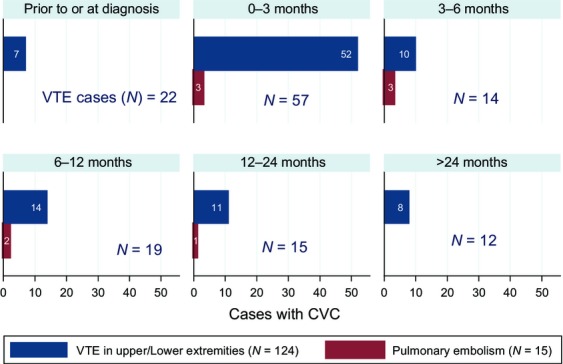
CVC presence among patients with VTE by time. CVC, central venous catheter.

Figure4 shows CVC-related VTEs with their time-to-event. A total of 139 patients had VTE including 124 with VTE in either upper or lower extremities and 15 with PE (PE: 8 patients and PE + DVT: 7 patients). Of these 124 patients, 102 (82.2%) had CVC placement within 3 months of the VTE. Among the 15 patients with PE, nine (60.0%) had CVC placement within 3 months of the PE. CVC-VTEs were frequent: they accounted for 83% (44/53) and 77.9% (67/86) of all events in ALL and AML, respectively. CVC-DVT events were responsible for 73.6% (39/53) and 73.2% (63/86) among ALL and AML groups, respectively; and CVC-PE events were responsible for 9.4% (5/53) and 4.7% (4/86) among ALL and AML groups, respectively.

**Figure 5:**
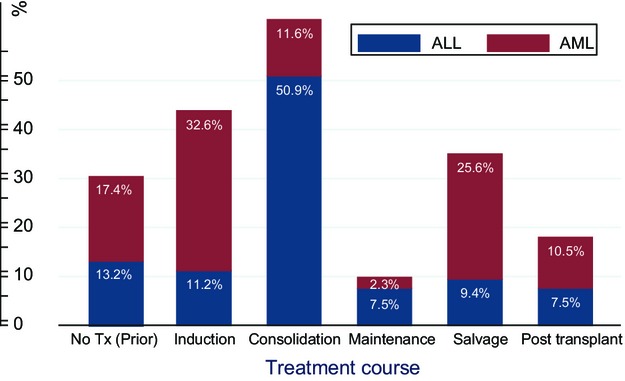
Occurrence of initial VTE according to treatment phase. VTE, venous thromboembolism.

**Figure 6:**
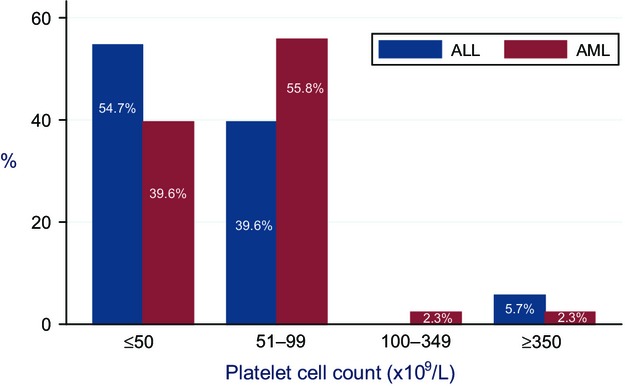
Platelet count at time of VTE diagnosis. VTE, venous thromboembolism.

Most ALL patients developed thrombosis during consolidation chemotherapy, whereas most AML patients developed VTE during induction chemotherapy (Fig.5). The mean time to event for ALL patients was 1.6 months (median time to event 2.4 months). In AML patients, the mean time to event was 2 months (median time to event 4.5 months). Most patients were thrombocytopenic when they developed VTE (Fig.6). The percentages of patients who had platelet counts of ≤50, 51–99, 100–349, or ≥350 × 10^9^/L at the time of acute VTE were 45.3%, 49.6%, 1.5%, and 3.6%, respectively. Around 55% of ALL patients and 39.6% of AML patients had platelet counts ≤50 × 10^9^/L at the time of VTE.

**Figure 7:**
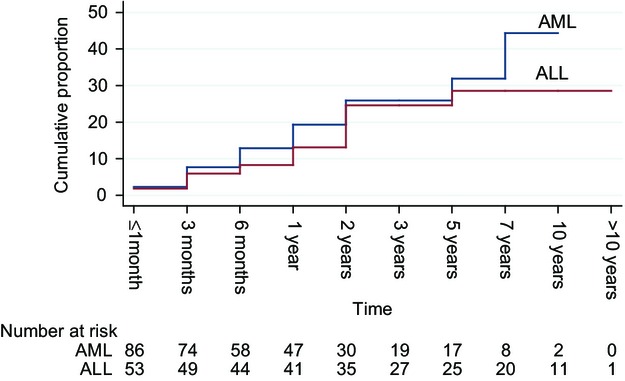
Cumulative proportion of recurrent VTE. VTE, venous thromboembolism.

**Figure 8:**
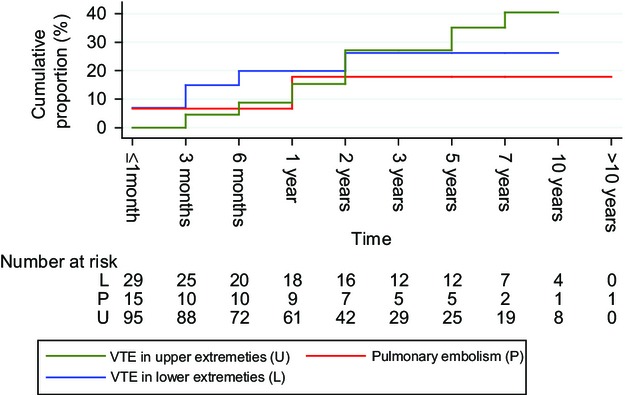
Cumulative proportion of recurrent VTE—according to anatomic location of first VTE event. VTE, venous thromboembolism.

The overall median follow-up period for all patients was 13.9 months (range, 0.03–123.9 months); during this time, VTE recurred in 20.7% (11/53) ALL patients and 18.6% (16/86) AML patients. The cumulative proportion of recurrent events for AML and ALL is shown in Figure7. Recurrence risk based on location of initial VTE is shown in Figure8. VTE recurred in upper extremities in 48.2% (13/27); in lower extremities in 25.9% (7/27); as PE in 18.5% (5/27) and in the CVC in 7.4% (2/27). Anticoagulation mainly with therapeutic low-molecular-weight heparin was used to treat VTE in 42/53 ALL patients (79%) and 38/86 AML patients (44%). It was continued for less than 1 month in 31% and 49% of patients with ALL and AML, respectively, and maintained for greater than 6 months in only 21% and 10% of patients with ALL and AML, respectively. Comparing patients with ALL versus those with AML, patients with ALL who developed VTE had a significantly longer time on therapeutic anticoagulation (mean time: 3.4 vs. 0.9 months, *P* < 0.0001). Acute leukemia patients with PE were treated with anticoagulation 2.3 months longer compared with those with DVT (mean: 3.9 vs. 1.6 months, *P* = 0.007). Interestingly, patients without a CVC had significantly longer times on therapeutic anticoagulation compared with those with CVC (3.0 vs. 1.5 months, *P* = 0.03). In the AML group, among 67 patients with CVC-related DVTs, two were exchanged, 14 were removed, and 18 were removed and treated with anticoagulation. In the ALL group, among 44 patients with CVC-related DVTs, four were removed and 10 were removed and treated with anticoagulation. No difference was found in the use of anticoagulation between those with and without CVC who developed PE, *P* = 0.319.

The use of anticoagulation appeared to have no bearing on recurrence: among ALL patients with VTE, the recurrence rate was 21% and 18% among those who received or did not receive therapeutic anticoagulation, respectively; for AML, it was 29% and 10%, respectively. Of note, at the time of recurrence, 0/11 ALL patients and only 1/16 AML patients were receiving therapeutic anticoagulation.

**Figure 9:**
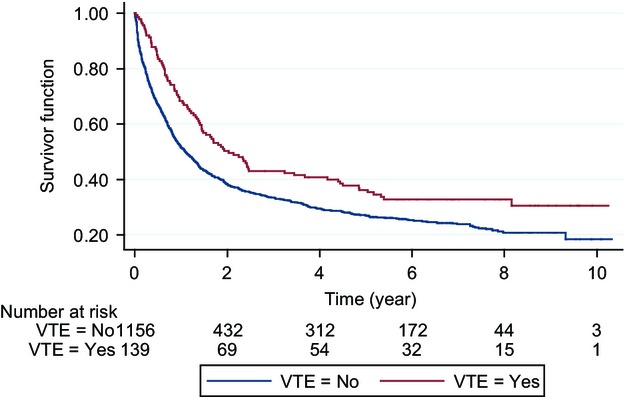
Kaplan–Meier survival estimates—patients with and without VTE. VTE, venous thromboembolism.

Over the course of the entire follow-up period, 73.7% of all patients died. None of the deaths were attributed to PE or VTE. Kaplan–Meier survival estimates for patients without and with VTE are shown in Figure9.

## Discussion

VTE is a common problem in patients with acute leukemia. We calculated an incidence density of 4.2 per 100 person-years in ALL and 3.4 per 100 person-years in AML. This is higher than those reported in previous studies 13,15,18,20–22. This may reflect the underutilization of pharmacological VTE prophylaxis at UTMDACC: <1% of ALL patients and <0.3% of AML patients received pharmacological prophylaxis during the period of analysis. In addition, previous studies may have underestimated VTE because data were derived from administrative or clinical databases, while our data were obtained from electronic medical records, an electronic pharmacy record system, and a clinical data base.

Many factors contribute to the prothrombotic state in acute leukemias 23–25. Factors in APL are characterized best: promyelocytes contain procoagulant-rich primary granules and express annexin II, which assembles the fibrinolytic apparatus on their surfaces 26,27. Exogenous factors include immobility, indwelling venous catheters, comorbidities (e.g., obesity, infection, renal disease, and pulmonary disease), ESAs, chemotherapy, and perhaps neutropenia 28,29. In this study, the relatively higher incidence of VTE in ALL patients almost certainly reflects that these patients received chemotherapy comprised of fractionated cyclophosphamide, vincristine, doxorubicin, and dexamethasone alternating with methotrexate and high-dose cytarabine 30. This regimen includes several notoriously thrombogenic agents, including asparaginase, which was part of the maintenance program in the hyper-CVAD regimen 31. Of note, 7/53 of ALL patients had received the thrombogenic agent asparaginase 32,33 prior to the development of VTE (four during hyper-CVAD maintenance program and three during salvage therapy), but asparaginase was used so infrequently that its impact on VTE rates cannot be calculated.

VTE can be the sentinel clinical event leading to a diagnosis of acute leukemia in up to 4% of patients 15. Of the 139 patients who developed VTE, five (4%) developed VTE prior to diagnosis and 13 (9%) were found to have VTE at leukemia diagnosis. These observations indicate that acute leukemia alone—separate from the effects of exogenous elements—is a risk factor for the development of VTE.

The risk of VTE is greatest within the first year following diagnosis and is especially high within the first few months, but this risk declines with time after diagnosis 13,15,18. Our data support these conclusions: 112/136 (80.6%) developed VTE within the first 12 months after diagnosis. VTE occurred during the initial hospitalization in 33% of ALL patients and 52% of AML patients.

Most VTEs developed in the upper extremities, and most of these were catheter-related. These results clarify the epidemiology of upper-extremity DVTs in patients with acute leukemia 23–25 and reemphasize that catheter-related thromboses are a major clinical problem. Efforts to develop innovative interventions for preventing catheter-related thrombosis are needed 34,35.

Cancer-associated VTE often recurs. Lee et al. found that almost 10% of cancer patients receiving optimal anticoagulation therapy had recurrent VTE during the 6 months of therapy 36. In our analysis, the VTE recurrence rate for both ALL and AML patients was about 20%, indicating that VTE often becomes a chronic problem. This large VTE recurrence rate supports the long-established conclusion that the treatment of cancer-related VTE is generally difficult. It also provides a focused point of intervention where improved therapeutics are needed and reemphasizes the importance of deducing why anticoagulation fails in this patient population. One obvious consideration is that therapeutic anticoagulation is often not achieved because of concerns that it will cause bleeding in patients with thrombocytopenia. Based on clinical guidelines, a platelet count <50 × 10^9^/L is considered a contraindication to anticoagulant therapy 7,37. No studies are available, however, to allow us to determine whether this guideline is appropriate in patients with acute leukemia who frequently become thrombocytopenic. Our data suggest that VTE recurrence rates could be decreased with better anticoagulation management during periods of thrombocytopenia.

The data presented, albeit extensive and thorough enough to establish several valid conclusions, require further prospective corroboration. Our study is limited because it is retrospective and descriptive. In particular, this design eliminates our capacity to elucidate the importance of inherited and acquired thrombophilia; to measure the impact of asymptomatic VTE, such as incidental PE or calf vein thrombosis; and to monitor and identify lethal PE. This latter deficiency is very important, as unrecognized lethal PE is common in many hospitalized patients 4–6, and it is impossible to establish the therapeutic index of pharmacological VTE prophylaxis without knowing for sure that thrombocytopenic leukemic patients are not dying from PE.

In conclusion, we observed that VTEs are common among patients with acute leukemia. They are more frequently associated with ALL than with AML, develop within a few months after leukemia diagnosis during periods of treatment-related thrombocytopenia, are usually CVC-associated, and often recur. Clinical tools to prevent catheter-related thrombosis and prospective data that can be used to optimize the therapeutic index of prophylactic and therapeutic anticoagulation in thrombocytopenic patients are needed.

## Conflict of Interest

Khanh Vu was formerly on the Speakers' Bureau of Sanofi. Michael H. Kroll is on the Scientific Advisory Committee of Aplagon Therapeutics and Boehringer-Ingelheim. The remaining authors have no conflict of interest to declare.

## Supporting Information

**Figure S1.** Kaplan–Meier survival estimates—according to VTE location among AML patients. **Figure S2.** Kaplan–Meier survival estimates—according to VTE location among ALL patients.
