# Supplementary material for: A retrospective study of venous thromboembolism in acute leukemia patients treated at the University of Texas MD Anderson Cancer Center
Source: Cancer Med. 2014 Dec 8;4(1):27–35. doi: 10.1002/cam4.332 (PMC4312115; doi:10.1002/cam4.332)
Supplement: Supplementary file 1 [file cam40004-0027-sd1.docx]

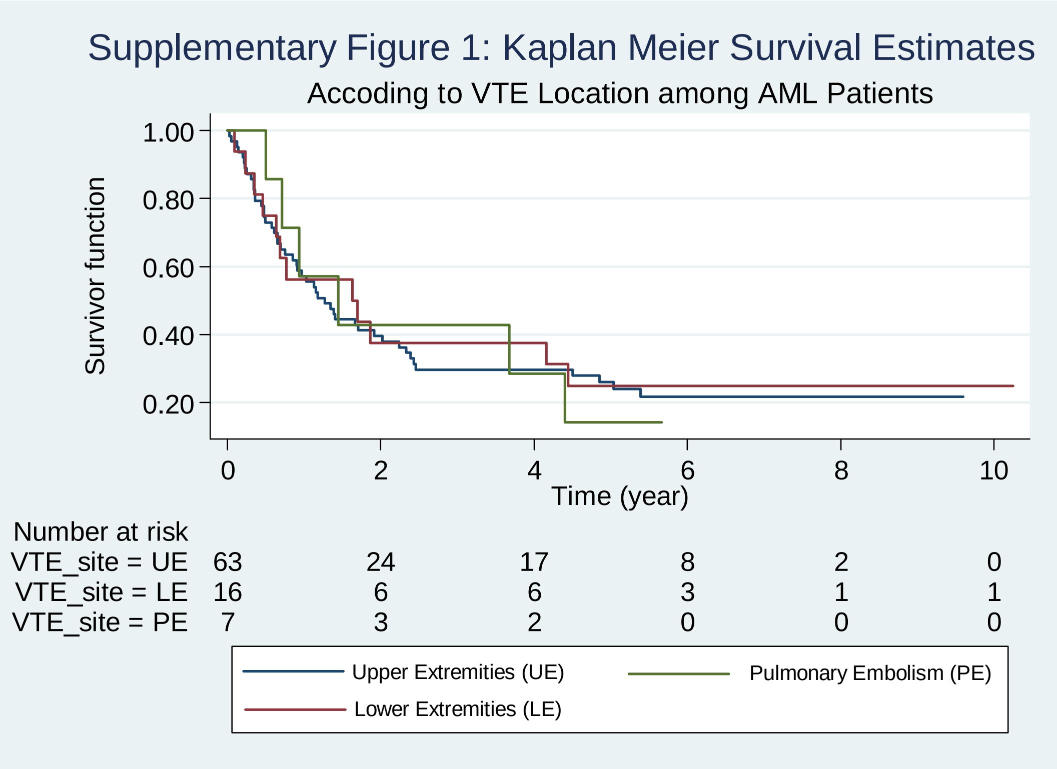
Supplementary Figure 1: Kaplan Meier Survival Estimates
Accoding to VTE Location among AML Patients
Survivor function
1.00
0.80
0.60
0.40
0.20
0
2
4
6
8
10
Time (year)
Number at risk
VTE_site = UE 63 24 17 8 2 0
VTE_site = LE 16 6 6 3 1 1
VTE_site = PE 7 3 2 0 0 0
Upper Extremities (UE)
Pulmonary Embolism (PE)
Lower Extremities (LE)

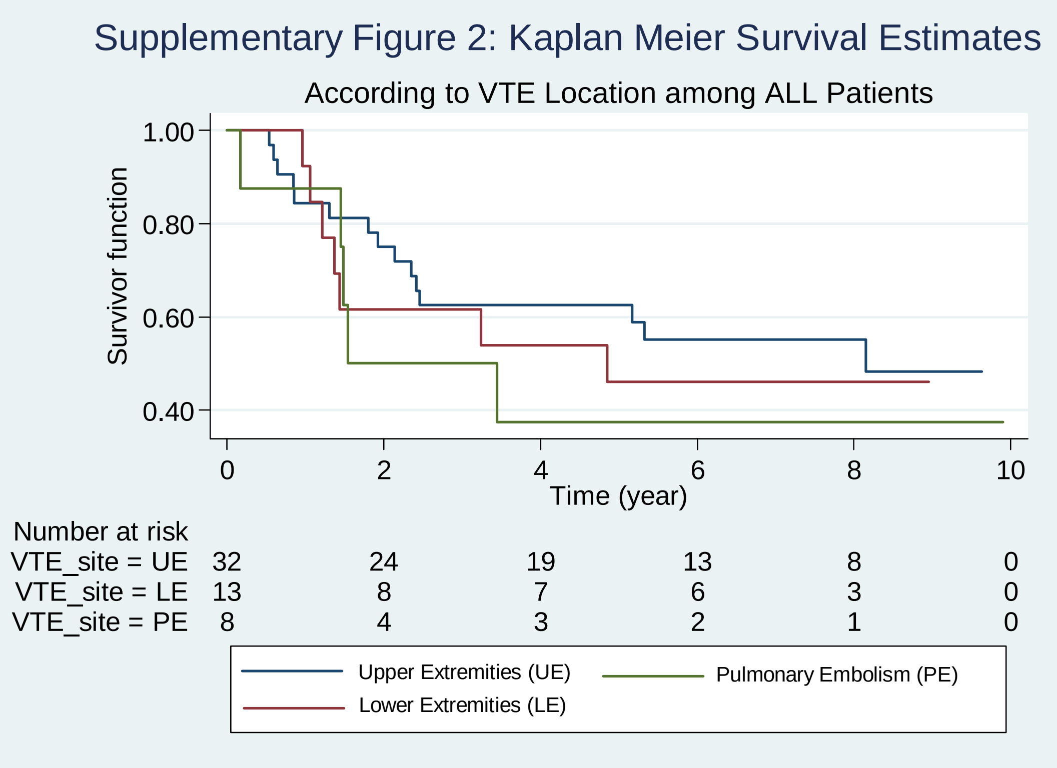
Supplementary Figure 2: Kaplan Meier Survival Estimates
According to VTE Location among ALL Patients
Survivor function
1.00
0.80
0.60
0.40
0
2
4
6
8
10
Time (year)
Number at risk
VTE_site = UE 32 24 19 13 8 0
VTE_site = LE 13 8 7 6 3 0
VTE_site = PE 8 4 3 2 1 0
Upper Extremities (UE)
Pulmonary Embolism (PE)
Lower Extremities (LE)
